# Altered mental status and low anion gap in a patient with sickle cell anemia: a case report

**DOI:** 10.1186/1752-1947-6-72

**Published:** 2012-02-20

**Authors:** Siddharth A Wartak, Reshma A Mehendale, Benjamin Freda, Ashish Verma, David N Rose

**Affiliations:** 1 Baystate Medical Center/Tufts University School of Medicine, Internal Medicine, 759 Chestnut Street, Springfield MA, 01199, USA

## Abstract

**Introduction:**

It is challenging to diagnose two coexisting medical conditions if the symptoms are overlapping. This is further confounded if the patient presents with an unexplained deterioration in mental status. A low anion gap or a zero anion gap is an uncommon clinical finding and has few differential diagnoses. This test therefore has important implications in correctly identifying underlying medical conditions.

**Case presentation:**

A 50-year-old African American male patient with sickle cell disease presented with refractory anemia, recurrent bone pains and encephalopathy. Routine testing failed to explain his mental deterioration. A laboratory finding of a low anion gap pointed in the direction of multiple myeloma as the underlying cause. This in turn led to an appropriate and timely course of treatment and clinical improvement.

**Conclusion:**

We present a very rare case of sickle cell anemia with coexisting multiple myeloma. This case sparks an interesting discussion on the anion gap, of which a clinician should be aware. It highlights the importance of the use of a verifiable anion gap in diagnosing medical conditions beyond the routine diagnosis of acid base disorders.

## Introduction

A 50-year-old patient with sickle cell disease presented with refractory anemia, recurrent bone pains and encephalopathy. Routine testing failed to explain his clinical deterioration and caused a diagnostic quandary. The clue to the diagnosis was his low anion gap, which revealed his underlying multiple myeloma and led to appropriate timely treatment and clinical improvement. This case illustrates the importance of the serum anion gap in diagnosing medical conditions beyond the routine diagnosis of acid base disorders.

## Case presentation

A 50-year-old African American man with sickle cell disease (SCD), transfusion-associated iron overload and stage III chronic kidney disease was admitted to our hospital with fever, shortness of breath and tachypnea. He was found to have bilateral lung crackles, leukocytosis and bilateral basilar infiltrates on chest radiography. Our patient reported a 15-pound weight loss and an increased frequency of bone pain over a period of six months. His medications included hydroxyurea and deferasirox. He was treated for painful sickle crisis and community-acquired pneumonia with analgesics, oxygen and antibiotics. He showed an initial improvement in clinical condition. He received repeated blood transfusions for his worsening anemia but did not show a subsequent increase in his hematocrit. His refractory anemia workup did not support any active bleeding or hemolysis. His reticulocyte count was 1.2% and his parvovirus B19 antibody assay was negative. A peripheral smear showed anisocytosis and Howell-Jolly bodies, but no schistocytes.

Our patient deteriorated four days after admission, with drowsiness, disorientation and slurred speech. A physical examination was unrevealing and a neurological examination showed no focal deficits. His electrolytes, blood urea nitrogen, creatinine, blood and urine cultures and brain imaging were indeterminate. His serum ammonia level was 81 μmol/L; he was started on lactulose with no clinical response. His opioid medications were decreased and he was given naloxone without improvement in his mental status. By the seventh day, he was stuporous. His family began to consider a 'comfort measures only' goal of care.

**Figure 1:**
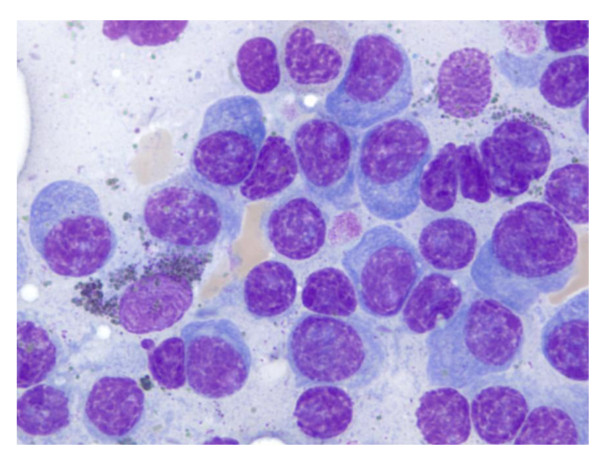
**Bone marrow biopsy showing plasma cells**.

This case was a diagnostic quandary. To decipher the cause of the refractory anemia, bone pain and change in mental status we focused on the surprising finding of a serum anion gap (AG) of zero (Table 1). His outpatient AG over the last six months had been between three and four. Further testing revealed that he had decreased serum albumin (2 g/dL), elevated serum protein (12.6 g/dL) and increased gamma globulin (7.5 g/dL). Immunofixation showed an immunoglobulin G monoclonal protein with kappa light chain specificity. Bone marrow analysis revealed 70% plasma cells, confirming the diagnosis of multiple myeloma (MM) (Figure 1). He was treated with dexamethasone (Decadron) and melphalan hydrochloride and made significant improvement, returning to his baseline functional status within 14 days.

**Table 1:** Laboratory results.

Test	Value
Hemoglobin	6.6 gm/dL
White cells	9.9 k/mm^3^
Platelets	235 k/mm^3^
Sodium	131 mmol/L
Potassium	4.7 mmol/L
Chloride	112 mmol/L
Bicarbonate	19 mmol/L
Glucose	107 mg/dL
Anion gap	0
Blood	21 mg/dL
Creatinine	1.5 mg/dL (Baseline 1.1 mg/dL)
Corrected calcium	9.5 mg/dL
Phosphorous	4.2 mg/dL
Magnesium	1.1 mEq/L
Ammonia	81 μmole/L
Total protein	12 gm/dL
Albumin	2 gm/dL
Alanine transaminase	42 units/L
Aspartate transaminase	43 units/L
Total bilirubin	0.8 mg/dL
International normalized ratio	1.2

## Discussion

Routinely measured cations and anions include sodium, potassium, chloride and bicarbonate. The sum of all circulating cations must equal the sum of all circulation anions [1]. The AG exists because the total unmeasured anions exceed the total unmeasured cations. Population studies using contemporary measurements of serum electrolytes suggest the normal range of AG to be between six and ten [1,2]. Furthermore, the concentration of potassium in the blood is usually negligible compared to that of sodium, chloride and bicarbonate. Therefore, the AG equals the sum of sodium ions minus the sum of the chloride ions and bicarbonate ions.

A low AG or a negative AG is uncommon in clinical medicine. There are very few clinical conditions associated with a low AG. Additionally, laboratory error may be a possible cause of a low AG finding. Thus, the test was repeated in our patient. The AG was still between zero and minus one. Normally, the majority of the serum AG is due to the sum of the anionic charges on circulating proteins. Albumin being the most abundant of all circulating proteins, hypoalbuminemia can result in a low AG. For each 1 g/dL decrement in the serum concentration of albumin, the serum AG decreases by 2.5 mEq/L [1]. In our patient, the serum albumin was 2.0 g/dL. It is therefore unlikely that this degree of hypoalbuminemia resulted in a zero or negative AG.

A rise in unmeasured cations including hyperkalemia, hypercalcemia and hypermagnesemia can also lower the AG. This usually remains clinically insignificant [1]. Severe intoxication with bromide, iodide or lithium can also lower the serum AG [1,3]. Our patient deteriorated four days after hospitalization and was thus unlikely to have been exposed to toxic levels of these substances.

In the evaluation of a verifiable low AG, one must also consider the presence of paraproteins. Overproduction of cationic monoclonal immunoglobulins, such as immunoglobulins G, can result in increased unmeasured cations. The serum AG in these patients can be below the normal range or, rarely, can even be negative [1,4,5].

Our patient presented with what appeared to be a sickle cell crisis. This likely masked his underlying MM and delayed the diagnosis. There are rare reports of SCD coexisting with MM [6]. The serum AG of zero led us to suspect and test for MM. Much of our patient's clinical presentation was initially thought to be caused by SCD. In retrospect, we must additionally consider the role of MM in his symptomatology. His refractory anemia can be explained by plasma cell infiltration of the bone marrow. The recurrent bone pain, thought to be secondary to a vaso- occlusive crisis, may be also explained by increased osteoclast activity, mediated particularly by interleukin 6 seen in MM [7]. The increased frequency of infection was likely a combination of SCD-associated autosplenectomy and immunodeficiency caused by the hypogammaglobulinemia of MM. Interestingly, the altered mental status remains unexplained despite a detailed workup. His encephalopathy cleared with treatment of his MM. Although somewhat speculative, his increased ammonia may have originated from his monoclonal plasma cells even in the presence of normal liver function [8-10].

## Conclusion

This case highlights the utility of a low serum AG in the diagnosis of MM. The presence of a zero or negative serum AG is an uncommon laboratory finding. As such, it has the potential to direct clinicians to evaluate for laboratory error, bromide intoxication or MM. This single test was key in closing the gap for an early diagnosis and for initiating prompt treatment of this potentially fatal illness.

## Consent

The patient described in our case report is deceased. Written informed consent for publication from the patient's next of kin could not be obtained despite all reasonable attempts. The case is important to public health and every effort has been made to protect the identity of our patient. There is no reason to believe that our patient's next of kin would object to publication.

## Competing interests

The authors declare that they have no competing interests.

## Authors' contributions

SW and DR analyzed and interpreted the patient data regarding the anion gap abnormality and diagnosed the underlying disease. SW, RM, AV, BF and DR were major contributors in writing the manuscript. All authors read and approved the final manuscript.
